# How to Measure Glycemic Variability? A Literature Review

**DOI:** 10.3390/medicina60010061

**Published:** 2023-12-28

**Authors:** Sandra Lazar, Ioana Ionita, Delia Reurean-Pintilei, Bogdan Timar

**Affiliations:** 1 First Department of Internal Medicine, “Victor Babes” University of Medicine and Pharmacy, 300041 Timisoara, Romania; sandra.lazar@umft.ro; 2 Department of Hematology, Emergency Municipal Hospital Timisoara, 300041 Timisoara, Romania; 3 Centre for Molecular Research in Nephrology and Vascular Disease, “Victor Babes” University of Medicine and Pharmacy, 300041 Timisoara, Romania; drdeliapintilei@gmail.com (D.R.-P.); bogdan.timar@umft.ro (B.T.); 4 Department of Diabetes, Nutrition and Metabolic Diseases, Consultmed Medical Centre, 700544 Iasi, Romania; 5 Second Department of Internal Medicine, “Victor Babes” University of Medicine and Pharmacy, 300041 Timisoara, Romania; 6 Department of Diabetes, “Pius Brinzeu” Emergency Hospital, 300723 Timisoara, Romania

**Keywords:** diabetes management, glycemic variability, glycemic fluctuations, coefficient of variability, time in range, continuous glucose monitoring systems

## Abstract

Optimal glycemic control without the presence of diabetes-related complications is the primary goal for adequate diabetes management. Recent studies have shown that hemoglobin A1c level cannot fully evaluate diabetes management as glycemic fluctuations are demonstrated to have a major impact on the occurrence of diabetes-related micro- and macroangiopathic comorbidities. The use of continuous glycemic monitoring systems allowed the quantification of glycemic fluctuations, providing valuable information about the patients’ glycemic control through various indicators that evaluate the magnitude of glycemic fluctuations in different time intervals. This review highlights the significance of glycemic variability by describing and providing a better understanding of common and alternative indicators available for use in clinical practice.

## 1. Introduction

For a long period of time, the hemoglobin A1c (HbA1c) level was considered the gold standard measurement in evaluating the quality of glycemic control and thus in evaluating the overall quality of diabetes mellitus (DM) management. However, recently, with the emerging, large-scale availability of continuous glucose monitoring systems (CGMS), a set of limitations associated with the single use of HbA1c such as the lack of quantification of glycemic value fluctuations or the lack of an obvious wave of the real number of hypoglycemic episodes led to an increase in the need of implementation of new valid indicators of glycemic control [1,2]. This was emphasized by the existence of patients with microvascular and macrovascular complications, despite maintaining an optimal range of HbA1c [3]. The large-scale use of CGMS allowed the use of novel, emerging indicators of glycemic control, not only the weighted average glycemic values (like in the case of HbA1c) counts but also glycemic variability (GV) [4].

GV represents the magnitude of the glycemia’s oscillations in a patient during a pre-defined interval of time. GV is a broad term comprising the variations in the glycemic values during very short intervals of time, like GV after events that can impact glycemia; during short or medium intervals of time; like GV for one day or long interval of times; or like differences between glycemic values at the same time of different days [5].

Currently, GV emerges as a valuable tool in evaluating the management of diabetes, showing that, on the one hand, GV is a predictor of diabetes complications and, on the other hand, a high GV represents a major burden in achieving the target values of traditional glycemic control parameters, like HbA1c. It is known that the amplitude of GV is positively correlated with the risk for development of all the diabetes chronic complications: neuropathy, retinopathy, chronic kidney diseases and macrovascular issues (such as coronary artery disease, peripheric artery disease, and cerebrovascular disease), this association being mainly mediated by the increase of inflammation and oxidative stress, which is a consequence of higher GV [6,7]. Moreover, increased GV was linked in several observations to a higher risk of mortality, an observation linked to both direct consequences of GV (i.e., by having a higher risk of severe hypoglycemic events or by emphasizing the risk of cardiac arrythmias) as well as based on indirect consequences (i.e., by increasing the risk of macrovascular diseases, major cardiovascular events, and other diabetes complications) [8]. Furthermore, a high GV leads to the impossibility to achieve optimal HbA1c target values without hypoglycemic events. A person with a higher GV will have a more frequent nadir glycemic value in the range of hypoglycemia compared to a person with similar HbA1c but lower GV [9].

Blood glucose values are homeostatic variables with a high degree of instability, even in short time spectrums, being influenced by several physiologic (i.e., glucose intake, emotional stress, or exercise) or pathologic (i.e., inflammation, infections or endocrine disorders) conditions [10]. This glycemic instability is more accentuated in patients with DM, especially in patients with type 1 diabetes. Having these important oscillations in short time intervals, evaluating GV was not feasible in clinical practice prior to the availability on a large scale of CGMS since the self-monitoring of blood glucose (SBMG) using the traditional methods via glucose meters lacked essential information regarding glucose oscillations between the measurements (the classical SMBG profile has seven daily measurements and usually is not performed by patients on a regular basis as a standard of care monitoring due to costs and discomfort). CGMS measure glycemia in short time intervals (usually at every 5 min), using a sensor that is inserted weekly or at two weeks subcutaneously, thus eliminating the need of fingerpicking and the costs of using blood glucose tests [11,12]. CGMS may reveal both the glycemic values and glycemic trends to the patient in real time, thus allowing him to act to improve glycemia and avoid hypoglycemia as well as to store the recorded values and thus to allow further analysis of the time-related glycemia, including here the analysis of GV [13].

Individualized glycemic targets based on several factors such as age, comorbidities, health-status and patient’s other general preferences are recommended [14]. For that, CGMS became a preferred method for monitoring blood glucose values for T1DM individuals, being primarily used for patients with recurrent acute diabetic complications (such as ketoacidosis), with higher prevalence of hypoglycemic events, or with associated health-states that impose a strictly glycemic control in a safe scenario or patients who cannot acquire the optimal value of HbA1c [5]. Even though the use of CGMS in T2DM is not as frequent compared to T1DM, a strong association of the impact of CV on diabetes management has been demonstrated in recent studies, thus the use of CGMS has also increased among these patients, especially in T2DM patients using insulin-based regimens [15].

Until recently, HbA1c was the only parameter for which a target value was established in order to achieve an optimal control of glycemic values, being set at a value below 7% (with slightly adjustments regarding elderly or pregnant patients) [14].

Since the introduction of CGMS, the American Diabetes Association (ADA) established the threshold values of the main indices that quantify GV and described their usage in clinical practice [14]. Firstly, in order to interpret the data collected by a CGM, it is necessary for a patient to wear it for a period of minimum 14 days, of which at least 70% of the time must be active. From this data, standard reports could be generated that illustrate a visual record of the blood glucose values and contain the main CGM metrics, such as mean of glycemic values, time in range (TIR), and coefficient of variability (CV) [14,16].

The understanding of GV has major clinical implications for DM patients. First is the evaluation of the risk of hypoglycemic events. A high GV, defined by drastic fluctuations of the blood glucose, could increase the risk of hypoglycemic events [17]. Secondly, GV has an impact on the quality of life in DM patients. It is known that optimal glycemic control is associated with better overall well-being. High glycemic fluctuations could lead to fatigue, dizziness, or mood swings that could decrease the quality of life [18]. Lastly, analyzing GV indicators could improve the overall DM treatment. Understanding of GV patterns could detect periods in which a patient tends to have hypoglycemic/hyperglycemic events, which could lead to treatment adjustments [19].

In this review we aim to summarize the indexes used to evaluate GV, how are they calculated and obtained, the advantages and disadvantages of each index, and the optimal scenario in which each GV index may be used.

## 2. Parameters Used to Evaluate Glycemic Variability

### 2.1. Standard Deviation

Standard deviation (SD) is the most basic index used to evaluate the GV. It describes the dispersion of all glucose measurements in a timeframe relative to the average glucose value in that timeframe. The SD is calculated as being the square root of the sum of squared differences between each value and the average, divided to the number of measurements minus one (Equation (1)). A higher SD is associated with an increased GV [11,20].

(1) $$Standard\;Deviation=\sqrt{\frac{\sum_{i=1}^{n}{\left(x_{i}-\bar{x}\right)}^{2}}{n-i}}$$

Equation (1): Standard deviation. *x_i_*—the *i*th glycemic value measured; $\bar{x}$—the average of glycemia; *n*—the number of glycemic measurements.

Simplified, one should imagine the SD as the average of the distances between each individual glycemia and the mean glycemia in the monitored timeframe. The SD has the advantage of evaluating all the glycemic variations in the defined timeframe and the advantage of being simple to calculate without the need of specialized software. However, from a clinical point of view, the SD has the main disadvantage that it reveals just the amplitude of glycemic oscillations; however, oscillations with the same amplitude have different clinical implications related to the starting point of the oscillation [21]. For example, if an increase with 60 mg/dL of glycemia from 50 mg/dL or from 300 mg/dL is recorded, in both cases the same SD will be obtained. However, from a clinical point of view, the oscillation from the first scenario has a much higher importance compared to the oscillation from the second scenario, a fact that cannot be assessed using the SD. Another drawback in using SD is that it can assess the GV just for the entire studied timeframe, thus making it impossible to evaluate GV during events of special interest (i.e., post-prandial or post-treatment GV) [22]. Moreover, since the SD counts all measurements in the monitored timeframe, the values close to the average may artificially decrease the SD, leading thus to underestimation of the magnitude of clinically relevant glycemic oscillations [22].

### 2.2. Coefficient of Variation

The coefficient of variation (CV) is obtained by dividing the SD to the average of the glycemic values (Equation (2)), thus being a GV index adjusted in relation to the average glycemia, resolving thus the clinical impact issue of using just SD to evaluate GV [17]. A higher CV is associated with higher GV. By evaluating the GV using CV, for the same absolute oscillation obtained at different average glycemia, a higher CV will be obtained for the oscillation associated with the lower average glycemia. The ease of calculation and ability to encompass the distribution of glycemic values to estimate the risk for hypoglycemic events make CV a reliable, large-scale, and often-used GV index. Besides resolving the clinical impact issue, the usage of CV has not demonstrated a great impact on diabetes control [17].

(2) CV=σx¯

Equation (2): Coefficient of variation. σ—standard deviation; $\bar{x}$—the average of glycemic values.

Current guidelines recommend achieving a CV lower than 0.36 (or 36% if expressed as percentage) considering that a CV lower than 0.36 is associated with stable glycemia, with respectively higher or equal to 0.36 denoting unstable blood glucose levels [14]. However, several studies set different threshold values for CV, below 0.33 (33%), suggesting that a stricter CV target reduces the risk of hypoglycemic episodes, especially in patients following insulin-based regimens or with oral hypoglycemic therapy such as sulfonylureas. Moreover, these targets can also vary depending on the demographic characteristics. For example, a study conducted in China including patients with T1DM and T2DM (divided by treatment type) set the threshold of CV at 0.33. This value was set based on the percentage of patients that achieved a target time in range value (above 70%) [23]. 

Even so, clinical analysis using T1DM patients highlights the importance of CV for diabetes management, observing that a lower CV is associated with a higher percentage of time spent in the range limit [24].

### 2.3. Mean Amplitude of Glycemic Excursions

The mean amplitude of glycemic excursions (MAGE) is a GV index that evaluates only the amplitude of important, clinically relevant variations, omitting from analysis any glycemic variations between one SD below and one SD above average glycemia. The MAGE index is calculated as the mean distances between consecutive nadirs and zeniths of blood glucose, which are lower than average minus SD respectively higher than average plus SD (Equation (3)) [25]. Thus, by removing the glycemic values close to the average from analysis, the MAGE index allows evaluation of the importance of the variations with clinical relevance, the variations having an amplitude higher than one SD [26]. The use of the MAGE index does not allow evaluation of the stability of the glycemic values, nor the time spent in hypo- or hyper-glycemia; however, it is mainly designed to provide insights regarding the extent to which glycemic excursions occur between fasting state hypoglycemia and postprandial hyperglycemia [27].

(3) $$MAGE=\sum \frac{\lambda }{n}\;if\;\lambda >\nu$$

Equation (3): Mean amplitude of glycemic excursions. *λ*—each blood glucose increases or decreases (nadir-peak or peak nadir); *n*—number of observations; *v* = 1 SD of mean glucose for 24 h period.

### 2.4. Continuous Overall Net Glycemic Action

The Continuous Overall Net Glycemic Action Index (CONGA) is a spectral analysis index that is obtained as the SD of the differences in glycemia between variable, pre-defined time intervals (spectrums) [22]. The CONGA index has the advantage of allowing evaluation of the GV for different pre-specified intervals, corresponding to different evaluated activities (Equation (4)). For example, by using CONGA-1 (for a 1 h interval time spectrum), the overall daily GV will be evaluated; by using CONGA-2 (for a 2 h time spectrum), the GV related to snacks for patients treated with regular insulin or meals for patients treated with rapid acting insulin analogues will be evaluated; by using CONGA-4 (a 4 h time spectrum), the GV related to meals in patients treated with regular insulins will be evaluated, respectively; by using CONGA-12 (a 12 h spectrum), data regarding the GV related to basal insulins may be analyzed [23]. The use of CONGA as a GV index has the main advantage of having flexibility regarding the time intervals for which GV is analyzed, thus fitting better to the individual clinical scenario that is evaluated [24].

(4) $$CONGA=\sqrt{\frac{{\sum \left(BG\left(t\right)-BG\left(t-n\right)\right)}^{2}}{k}}$$

Equation (4): Continuous overall net glycemic action. *BG*(*t*)—the blood glucose value at time t; *BG*(*t* − *n*)—the blood glucose value at time *t* − *n* (n is the time interval); Σ—the sum of the squared differences between consecutive blood glucose values; *k*—the time interval between blood glucose measurements.

### 2.5. The Mean of Daily Differences

The mean of daily differences (MODD) is the currently accepted standard index for evaluating the between-days GV [25]. The MODD is calculated as the mean absolute differences between glycemia at the same time in two consecutive days. The MODD is mainly used to evaluate the predictability of different glucose therapies and regimens (Equation (5)) [26]. A higher MODD value is associated with decreased treatment predictability and increased glycemic uncertainty [25]. An increased MODD leads to more difficult assignment of achieving a low HbA1c in safety conditions: when the average glucose is to be decreased in a patient with increased GV between days, there is a significantly increased risk for developing hypoglycemic events [27].

(5) $$MODD=\frac{\sum \left|BG\left(t\right)-BG(t-1)\right|}{n}$$

Equation (5): The mean of daily differences. *BG*(*t*)—the blood glucose value at time *t*; *BG*(*t* − 1)—the blood glucose value at the previous time point; Σ—the sum of the absolute differences between consecutive blood glucose values; *n*—the number of blood glucose measurements taken in a day.

### 2.6. J-Index

The J-index is a hybrid index, which equally evaluates both hyperglycemia as well as GV. The value of the J-index is equally increased by the increases of GV (measured using the SD) as well as by the increases in the average glycemia. Thus, the J-index may be considered a vector of glycemic imbalance, a condition described by both hyperglycemia and high GV [28].

### 2.7. Time in Range

TIR is not a per se index of GV but may indirectly provide valuable information regarding both the quality of glycemic control as well as the degree of GV. TIR emerges as a valuable measure of the quality of glycemic control with the increased access in the future to CGMS; it is possible that TIR will undertake hemoglobin A1c as the standard of care method used to assess the quality of the glycemic control and diabetes treatment’s efficacy [29]. Current guidelines are emphasizing the role of using TIR in clinical practice, pointing to its advantages over HbA1c, which is currently the standard of care method to evaluate glycemic control and treatment efficacy [30]. While HbA1c represents a weighted average value of glucose values up to the last 90 days, it cannot evaluate the glycemic oscillations; thus a good HbA1c may be obtained as an average of optimal values or as an average of extreme, undesired glycemic values (hyper- and hypo-glycemia).

### 2.8. Low Blood Glucose Index

The low blood glucose index (LBGI) is an indicator of GV that measures the area under the curve when blood sugar drops below a predetermined range (Equation (6)). For LBGI calculation, hyperglycemic episodes are excluded. The LBGI is considered to be a predictive indicator for determining the patients who are at risk of developing hypoglycemic episodes, having a positive impact on glycemic control, especially during nighttime [29,30,31].

(6) $$LBGI=\frac{1}{n\times \sum \left(10\times fbg2i\right)},\;where\;fbgi=min\;(0,\;1.509\times (\log\left(BGi\right)1.084-5.381)$$

Equation (6): Low blood glucose index.

### 2.9. High Blood Glucose Index

The high blood glucose index (HBGI) is an indicator that quantifies the risk of hyperglycemic episodes by measuring the area under the curve when the blood glucose value is above a predetermined value (Equation (7)) [11]. The HBGI and LBGI have been shown to be predictive indicators for subsequent glycemic events, but their applicability has not been validated [31,32].

(7) $$HBGI=\frac{1}{n\times \sum \left(10\times fbg2i\right)},\;where\;fbgi=max\;(0,\;1.509\times (\log\left(BGi\right)1.084-5.381)$$

Equation (7): High blood glucose index.

### 2.10. Average Daily Risk Rates

Average daily risk rates (ADRR) is an indicator of the daily risk of occurrence of hypoglycemic and hyperglycemic excursions. To calculate the ADRR, it is necessary to measure the glycemic values over an interval of at least 14 days, and it represents the daily sum of the highest or the lowest glycemic value outside the TIR, averaged over the daily amounts. In case there are no glycemic values outside the TIR on a certain day, that day will be counted as zero [32]. Thus, the ADRR is divided into three risk categories: low risk, moderate risk, and high risk [33]. The ADRR has been shown in studies to be a reliable predictor of excessive glucose levels.

### 2.11. M Value

The M value is a hybrid index of both GV and mean of glycemic values, providing an overview of glycemic behavior. The assessment of diabetes management is by comparing glucose fluctuations by a value 6.6 mmol/L—considered to be ideal. In healthy individuals, M value is zero, but it rises with higher glycemic excursions. It is considered to have a greater clinical impact due to its higher increase in the presence of hypoglycemic episodes compared to hyperglycemic ones. The calculation requires the sum of the average value of the logarithmic transformation of the deviation from the reference value over a 24 h period with a magnitude correction factor [28].

## 3. Discussion

### 3.1. Examples of Clinical Scenarios

To emphasize the limitations of HbA1c and understand the clinical applicability of GV indices, a series of clinical scenarios have been proposed.

It can be considered that there are two DM patients with the same HbA1c level. For the first patient, the HbA1c level was obtained from close and constant blood glucose values, while in the second case, HbA1c was obtained from distant blood glucose values. Thus, if the VG indices are calculated, the first patient will have a lower CV and SD, respectively, and a higher TIR, suggesting better glycemic control compared to the second patient, despite obtaining the same HbA1c value. Being just a weighted average, evaluation of glycemic control only by HbA1c cannot offer information about the dispersion of glycemic values.

However, the assessment of GV must consider all the main indicators because the independent analysis of a single indicator could generate errors.

For example, consider if there are two patients, patient A and patient B, and patient A has a lower HbA1c value compared to patient B but GV assessment detects the same SD value. In this case, evaluation of glycemic values only by SD would generate the hypothesis of a similar GV. Thereby, evaluation of CV and TIR is needed, having an increased clinical importance. In this case, patient A will have a higher TIR compared to patient B, suggesting the existence of an association between HbA1c and TIR.

Moreover, assessment of short-term GV is a major component for quantifying glycemic control. The use of CGMS allowed the introduction of the MAGE index, currently the “gold standard” for assessing short-time with-day GV indicating the degree of stability of glycemic values.

Therefore, the evaluation of glycemic control in two patients with the same HbA1c and similar values of CV and SD indicates similar glycemic stability between patients, with similar metabolic control. However, the value of the MAGE indicator is higher in the first patient, suggesting that he will have a higher risk of hypoglycemic or hyperglycemic episodes, in parallel with a lower TIR value. The subsequent calculation of the LBGI and HBGI will indicate in which direction the risk of dysglycemic values is directed (hypoglycemia vs. hyperglycemia).

The assessment of the degree of dispersion of glycemic values is principally made through three indicators, SD, MAGE, and MODD. The MODD calculates the absolute difference of glycemic values obtained in the same period of the day, in two consecutive days, thus helping to discover individualized patterns.

So, for two patients with similar values of HbA1c, SD, CV TIR, and MAGE but with a different MODD value, the first patient having a lower MODD value compared to the second one, it can indicate that there is a higher probability of discovering patterns and thus generating a successful intervention for the first patient.

### 3.2. Advantages and Disadvantages of Glycemic Variability Indicators

TIR is becoming an emerging standard in the evaluation of DM management [16]. However, it is only partially associated with the phenomenon of GV. Most of the time, a lower TIR is associated with increased GV, but there are particular situations in which a patient with a reduced TIR can have a reduced GV, for example, a patient with stable blood glucose values but with increased average of glycemic levels without significant oscillation. In this case, the patient will have a reduced SD, CV, and MAGE level in parallel with a reduced TIR percentage, given by the fact that the patient has predominantly high blood sugar levels.

CV and TIR are presented automatically by the report from the sensor. By contrast, the other GV indices need to be calculated with third-party software. The comprehensive evaluation cannot be done only by TIR and CV, so in clinical situations that impose difficulties, all the indexes must be used together because each one offers different and complementary information regarding the assessment of glycemic control.

Although they are not used on the same scale as the previously mentioned indicators, the LGBI, HBGI, and ADRR require particular attention because they are providing important insights of GV by quantifying the risk of hypoglycemia and hyperglycemia [11]. Their use allows dysglycemic assessment, along with providing a numerical representation of dysglycemic risk, thus allowing therapeutic interventions that can be critical for patient safety [31]. Their limitations are based on the complexity of the calculation and on the individual variability in the response to insulin action but also on the fact that there is no universal threshold value of these indices, making it challenging to establish universal applicability standards.

The J-index and M-values are both hybrid indicators used to assess glycemic behavior.

The J index offers a quantitative measure of GV, incorporating both hypoglycemic and hyperglycemic events, that accounts for the rate of glucose oscillations, giving insights about the rapidity of changes in blood glucose values [32]. The important limitations of the J index are represented by the complex calculation and the challenges in interpretation, due to the fact that there is no universally agreed threshold for optimal/suboptimal GV.

On the other hand, the M value is not a sole indicator for GV. Even though it provides an overview on glycemic behavior, its clinical applicability was not demonstrated [28].

### 3.3. Findings from the Literature

Recent literature data have demonstrated the importance and applicability of GV in current medical practice [33].

Experimental studies mentioned that hyperglycemic spike after a glucose tolerance test and glucose fluctuations are themselves predictors for macrovascular complication, such as coronary artery disease, being emphasized that intermittent hyperglycemia has a damaging effect on blood vessels compared to chronic hyperglycemia [34,35]. This was later observed in clinical studies such as the Coronary Artery Calcification in Type 1 Diabetes (CACTI) study that included young patients with type 1 diabetes mellitus (T1DM) and that observed that higher GV, measured through SD, was positively correlated with coronary artery diseases [36].

On the other hand, GV had demonstrated its impact even in type 2 diabetes mellitus (T2DM). A clinical study that included over 100 type 2 diabetic patients indicated that GV, measured by SD and MAGE, was an independent risk factor for diabetic retinopathy, regarding HbA1c value [37].

The need for new DM management metrics beyond HbA1c leads to the development of TIR targets. TIR also demonstrated its impact on providing clinical guidance for glycemic management, proving that there is a relationship between HbA1c and TIR [16,38]. Moreover, several studies including a cross-sectional study that used Diabetes Control and Complications Trial (DCCT) data demonstrated that there is a strong correlation between TIR and the risk of occurrence of DM complications [39].

However, future research is needed for actively exploring the impact of GV on long-term diabetes complications in order to develop treatment strategies to minimize it for improving glycemic control and overall DM management.

## 4. Conclusions

Considering that GV is independently associated with an increased risk of both short- and long-term DM complications, including hypo- or hyper-glycemia and micro- and macro-vascular events and thus significantly impacting the overall prognosis of a patient with DM, the use of GV indicators should be the standard of care in evaluating the quality of DM management, in addition to the traditional parameters, like HbA1c, self-monitored blood glucose or number, and intensity or duration of hypoglycemic events. Furthermore, GV has been associated with quality of life and healthcare costs.

Further research is needed to establish standardized thresholds for indicators of GV and to explore the impact of interventions targeting GV on patient outcomes. In addition, the development of user-friendly tools and technologies for continuous glucose monitoring and analysis will facilitate more accurate and convenient assessment of glucose variability in clinical settings.

By understanding and addressing GV, healthcare professionals can strive for improved glycemic control, improved patient outcomes, and ultimately a better quality of life for DM patients.

## Data Availability

Not applicable.
